# Classroom perception in higher education: The impact of spatial factors on student satisfaction in lecture versus active learning classrooms

**DOI:** 10.3389/fpsyg.2022.941285

**Published:** 2022-09-27

**Authors:** Shitao Jin, Lei Peng

**Affiliations:** ^1^School of Architecture and Urban Planning, Huazhong University of Science and Technology, Wuhan, China; ^2^Hubei Engineering and Technology Research Center of Urbanization, Wuhan, China

**Keywords:** classroom perception, learning environment, student satisfaction, lecture classroom, active learning classroom

## Abstract

Driven and influenced by learning theory and information technology, the form of the classroom environment in higher education is constantly changing. While traditional lecture classrooms focus on efficient learning modes and economical space layouts, active learning classrooms focus more on active learning psychology and adaptive space perception. Although existing studies have explored the development of educational and technological domains in the classroom, a comparative study of these two classroom environments and students’ learning perceptions has not been conducted. Hence, using a questionnaire, this study collected subjective perception reports from 316 students in traditional lecture classrooms versus active learning classrooms. By analyzing Likert scales of student satisfaction in two classroom environments, the study found the following: learning support dimensions in both classroom environments required improvements; space and furniture perception, physical and decorative environment are critical factors in improving students’ perceptions in the lecture classroom; space perception is the critical factor in enhancing students’ perception in the active learning classroom. These findings can serve as good references and useful insights for future classroom design and optimization to build enriched and inclusive learning environments to help students gain a more positive perception of learning.

## Introduction

Since the 1990s, with the development of learning theory and educational technology, researchers have gained a new understanding of the nature of learning, and teaching and learning approaches have experienced diverse and profound changes (Beetham and Sharpe, 2013). As the primary place for teachers to teach and students to learn, classroom space garners increasing attention (Harrison and Hutton, 2013). Many countries and regions are actively investigating new classroom spaces, including Innovative Learning Environments (OECD, 2013), Technology-enhanced Learning Environments (Brooks, 2011), and Next Generation of Learning Space (Wilson and Randall, 2022). Although these new classroom spaces vary, they all adhere to the same theoretical principles of being student-centered and encouraging collaborative, inquisitorial, and contextualized learning (Marais, 2011). In addition, they all combine spatial design and active learning pedagogy (Horne et al., 2012), and are equipped with a variety of flexible furniture and equipment (Fisher and Newton, 2014) to enhance student learning outcomes (Radcliffe, 2008). These new classroom spaces are referred to as active learning classrooms (Talbert and Mor-Avi, 2019).

Traditional lecture classroom spaces have long been influenced by behaviorist theories (Merriam et al., 2007), with teaching layouts and technologies that primarily serve teacher-led classroom lectures (Park and Choi, 2014) and create a controlled and orderly learning environment. As a new learning space distinct from lecture classrooms, active learning classrooms integrate constructivist learning theory^1^ and information-age technological devices into the classroom environment, emphasizing the need for cooperation among students and active knowledge construction (Ashworth et al., 2004), and creating a diverse, flexible, and intelligent learning environment (Hacisalihoglu et al., 2018). Previous studies have demonstrated that active learning classrooms have a more significant impact on students’ learning outcomes than lecture classrooms (Brooks, 2011; Baepler et al., 2014; Park and Choi, 2014; Byers et al., 2018b; Lo and Hew, 2021). However, lecture classrooms remain the prevalent classroom space on university campuses (Zhong Qiquan, 2015; Soneral and Wyse, 2017), as lectures are the primary teaching method in introductory undergraduate classes, and the proportion of seminars will gradually increase as the grades go up to the graduate level (Merriam et al., 2007). In addition, the larger *per capita* floor space, and higher costs for furniture and technological equipment compared to lecture classrooms limit active learning classrooms’ construction (Soneral and Wyse, 2017). Conversely, lecture classrooms have a larger student capacity and are more affordable and logistically easier to manage, making them an indispensable learning space in the present day.

Owing to their single spatial layout and limited technological equipment (Stoltzfus et al., 2016), traditional lecture classrooms do not provide enough support for learning methods such as collaborative, exploratory, and active learning emphasized by learning theories such as cognitivism and constructivism (Painter et al., 2013), resulting in poor user experience and students satisfaction. A central issue for current lecture classrooms is improving student satisfaction through targeted and economical renovation design and promoting the construction of student knowledge in problem identification, analysis, and problem-solving while maintaining a high volume and low cost of classroom space. Furthermore, as active learning classrooms are relatively new, research in this field is in the early stages, demonstrating the general emphasis on practice rather than research (Temple, 2008; Ellis and Goodyear, 2016). Diversified space layouts and constantly enriched technical equipment require active learning classrooms to keep up with constant development and improvements. Student satisfaction and the spatial attributes that influence it in active learning classrooms have not been investigated to date. This study surveyed students in lecture and active learning classrooms at a university in China to identify indicators of satisfaction with the learning space. The following research questions were posed:

1. In terms of the current space in traditional lecture classrooms and active learning classrooms, which elements are students most satisfied with? Which elements are the least satisfying?
2. Has the change in the spatial environment from lecture to active learning classrooms affected student satisfaction? What impacts and changes have resulted?
3. What spatial attributes affect students’ overall satisfaction with lecture and active learning classrooms? Does sex, subject major, grade, and other personal characteristic affect students’ spatial perception? How can the lecture classroom be improved with low cost and low intervention? How can the active learning classroom be further optimized?

## Literature review

### The influence of learning theory on the learning environment

As the dominant school of psychology in the 1950s, behaviorism influenced the instructional design theory of the time (Schunk, 2011). It emphasizes learners’ passive acceptance and continuous stimulation of knowledge so that learners can respond and adapt effectively (Winn, 1990). The traditional lecture classroom is deeply influenced by behaviorism, forming a spatial basis for teachers to unilaterally impart knowledge to students, which emphasizes the structured order of the classroom and the memory and recitation of knowledge (Ashworth et al., 2004). Under the influence of this learning theory, the traditional lecture classroom does not pay attention to the individual differences of learners and the diversity of knowledge content, but its efficient and simple learning process affords it an important position in the historical development of the learning environment.

In the late 1950s, learning theory gradually shifted from behaviorism to cognitivism dominated by cognitive science. Cognitivism pays more attention to deep-seated internal cognitive processes, such as thinking organization, problem-solving, language logic, etc. (Davis, 1976). The instructional design theory, focuses more attention on learners’ psychological activities and learning cognition, and emphasizes learners’ active participation and thinking in knowledge transmission (Ertmer and Newby, 1993). However, the changes brought about by cognitivism are more reflected in teaching strategies and learning methods, and the classroom environment has not changed much.

The emergence of constructivism breaks the objectivism theory based on behaviorism and cognitivism, that the learner acquires knowledge from the outside world (Jonassen, 1991a). Constructivism assumes that the learners construct an interpretation of knowledge itself through personal experience and activities. Although constructivism and cognitivism both regard learning as a psychological activity, the former believes that the learner’s mind is a filter that can filter the knowledge of the real world and form its unique ideological reality, rather than just a reference to the real world (Jonassen, 1991b). In addition, constructivism not only regards learners as active participants in knowledge but also encourages them to develop a unique understanding and individualized use of information (Duffy and Jonassen, 1991). Therefore, the classroom environment influenced by this learning theory has also changed into a more active and collaborative learning environment (Jamieson, 2003; Brown and long, 2009). Classroom space is not only an environment for personal learning but also a place for collaboration among learners to promote innovation in communication (Simonton, 2000). The learning environment is not only more diverse and flexible but also more situational and practical to meet the needs of authenticity and interdisciplinary research (Sawyer, 2006; Horne et al., 2012). With the development of information technology, the idea of constructivism is also easier to realize in the classroom. For example, interactive display devices provide teachers with a variety of teaching methods to promote diversified classroom research (Brown and long, 2009). Internet technology supports students to access online learning materials and realize multidisciplinary real-time interactive communication (Colace et al., 2003).

### Traditional lecture classrooms’ historical background and spatial design

**Figure 1:**
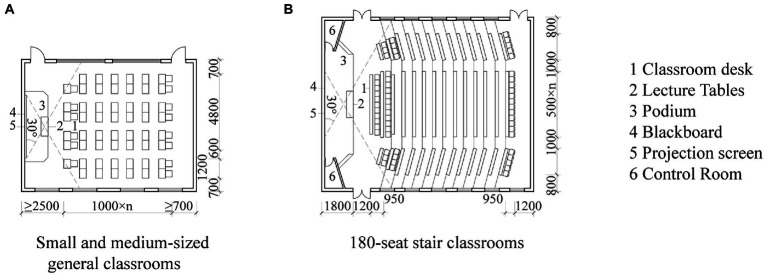
Examples of standard lecture classroom layouts presented in the Architectural Design Sourcebook (China Construction Industry Press, 2019). Reproduced with permission of China Construction Industry Press.

The large-scale construction of traditional lecture classrooms took place during the period of modernism architecture after the Second World War, much of which is still in operation today and has continued in subsequent classroom construction (Dovey and Fisher, 2014). In lecture classrooms, fixed teaching facilities, such as a raised podium area and regularly spaced parallel desks and chairs (Park and Choi, 2014), occupy the entire spatial environment (Reynard, 2009). The teacher’s podium is at the center of the students’ eyeline, and furniture and equipment are arranged so that the teacher controls the entire learning environment, emphasizing standardized education and efficient transfer of knowledge. This standardized classroom floor plan has also become a common form of lecture classrooms in universities in various countries (Baker, 2012). Byers et al. (2018a) found that in lecture classrooms, where teachers and students tend to be influenced by the format of the classroom space, there is a higher probability of teacher-centered instruction (Byers et al., 2018a). Dovey and Fisher (2014) argued that the fixed spatial layout of the lecture classroom prevents professors from employing more diversified pedagogies and impedes students’ initiative in learning, which is advocated by innovative teaching philosophy. In China, lecture classrooms continue to use the fixed layout and parallel arrangement of spatial patterns. In the 1950s, China’s educational ideology was largely based on national construction and the urgent need for standardized, unified education to train engineering and technical personnel. The educational environment tended to pursue a steady, atmospheric style (Ji, 2019). Figure 1 shows that lecture classrooms in Chinese universities are designed based on the Architectural Design Sourcebook (China Construction Industry Press, 2019), which presents standardization and uniformity. Classroom space is designed mainly to accommodate many students based on the learning concept of silent classroom behavior (Linhai, 2016), in which teachers teach and students listen. This forms a seedling layout, with teachers as the classroom leaders and students arranged in parallel and extending backward in sequence (Liao Shiyan, 2019). This learning space continues to be used until the present.

### Active learning classrooms’ practical and theoretical research

Active learning classrooms are student-centered and use various digital technologies and information interaction devices (Dori and Belcher, 2005) to create a learning space that not only meets the needs of constructivism teaching and innovative practice application (Marais, 2011) but also meets the needs of personalized and adaptable multi-oriented cultivation (Brooks, 2011). Existing research on this new learning environment has focused on two main aspects: the construction of active learning classrooms, and the effectiveness and evaluation of active learning classrooms.

First, the practice of active learning classrooms began in the 1990s with the SCALE-UP (Student-Centered Active Learning Environment for Undergraduate Programs) project at North Carolina State University. This project used a collaborative round-table spatial layout in a classroom for the first time and included extensive technological equipment to increase the flexibility and maneuverability of the classroom, allowing students to interact and collaborate in small groups and have direct access to the content (Beichner and Saul, 2003). This created a shift from the previous teacher-centered approach to a student-centered environment and laid the foundation for active learning classrooms (Beichner et al., 2007). The Massachusetts Institute of Technology proposed the TEAL (Technology Enables Active Learning) model in 2003. This classroom model uses advanced visual media simulations and personal response systems to enhance student collaboration and learning (Dori et al., 2003). The unique design of the classroom space builds a visual understanding of content concepts and principles for students, which improves attendance and lowers student failure rates (Dori and Belcher, 2005). The University of Minnesota, influenced by SCALE-UP and TEAL, proposed the PAIR-up (Pedagogy-rich, Assess learning impact, Integrate innovations, Revisit emerging technologies) model of active learning classrooms in 2006 (Whiteside, 2009). This model focuses on improving the flexibility of active learning classrooms, adopting the most popular wall system technology at the time, using new building materials, such as detachable walls and spliceable floors, allowing teachers and students to experience new classroom designs, and utilizing various teaching strategies. The University of Iowa created the TILE (Transform, Interact, Learn, Engage) model of active learning classroom space in 2012, which combines teacher instructional methodologies and classroom space design and offers a variety of technological devices (Horne et al., 2012). In addition, TILE includes three classroom sizes that can accommodate 27, 54, and 81 students. The multiple space sizes improve the adaptability of the classrooms and can meet the specialized needs of various disciplines (Florman, 2014). Henceforth, many countries and universities have explored active learning classrooms and developed their models, such as Engaged, Active Student Learning (EASL) classrooms (Gatlin et al., 2021), which were based on the TILE classroom space model and incorporated the opinions of local teachers and students. Many universities in China, including Sichuan University (Sichuan University, 2022), Huazhong Normal University (Huazhong Normal University, 2022), and Huazhong University of Science and Technology (Huazhong University of Science and Technology, 2022), have investigated active learning classrooms in recent years. These classroom spaces are developed and built based on SCALE-UP prototypes.

Second, in the evaluation studies of active learning classrooms, the main focus is on the subjective evaluation of learning space users, a process that includes both scientific and anthropological reflections on the elements of assessment (Hadjri and Crozier, 2009). Parsons (2016) found through interviews and classroom observations that the abundant spatial layout of active learning classrooms can positively influence students’ classroom communication, and the diverse furniture design can accommodate different learning modes such as individual learning and group work, and can be arranged more user-friendly according to the instructional content. Using a questionnaire to survey students, Young et al. (2021) found that flexible classroom space design improved adaptability to a range of teacher-and student-led activities. Through multiple quasi-experimental studies, Brooks (2011, 2012) found that students’ learning outcomes, progress, and learning behaviors improved in active learning classrooms with richer spaces and technology than in traditional classrooms. Based on MIT’s TEAL learning space, Dori et al. (2003) found through a comparative experiment that the diverse spatial environment and advanced technology configuration of active learning classrooms led to better conceptual understanding than in traditional classrooms, and that most students had positive attitudes toward active learning classrooms (Dori and Belcher, 2005). In addition, research on this learning environment does not stop at universities and academia; many countries have established departments to evaluate the performance of active learning classrooms. The Higher Education Funding Council (HEC) has established 10 guidelines for assessing the inclusiveness and diversity of this learning space (Pearshouse et al., 2009), and the Next Generation Learning Space Study Group, established by the Australian Government and universities, has provided a systematic review and summary of the future development for this learning space to be more flexible, open, and inclusive (Wilson and Randall, 2022).

## Classroom space at the surveyed university

### Traditional lecture classrooms

The university participating in this study was founded in 1952 in the central western regions of China (university H), founded after the founding of the People’s Republic of China and is typical of post-liberation universities. The campus’s construction and development cover a long-time span, demonstrating changes in the design models and standards of Chinese university educational buildings. There are 293 lecture classrooms, which are located in various public academic buildings and specialized departmental buildings on the main campus. Table 1 shows that the traditional classroom spaces of this university have the following attributes and characteristics. First, the student capacity of the classrooms ranges from 30 to 250, and the *per capita* use area is about 1 square meter. Second, the function of these spaces is divided into teacher and student areas, with teacher areas raised 200 mm and student areas consisting of rows of fixed seats and corridors. Third, all traditional lecture classrooms use an inline space layout, which is simple to design and economical to build. Fourth, classrooms with fewer than 60 students utilize two-person desks and chairs, and classrooms with more than 60 students use fixed continuous rows of long desks and seats, with the seats in the front row connected to the desks in the row behind. Fifth, all traditional lecture classrooms are outfitted with blackboards, projectors, desktop computers, and microphones that can meet the fundamental demands of teachers in class.

**Table 1:** Current status of lecture classroom space at university H.

Size	36 people	50 people	60 people	60 people	110 people	240 people
Space layout	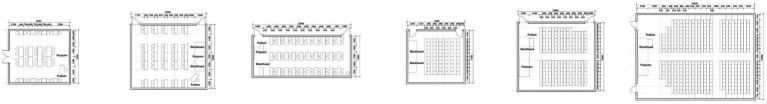					
Functional division	Three zones two corridors in straight rows	Three zones two corridors in straight rows	Three zones two corridors in straight rows	One zone two corridors in straight rows	Two zones three corridors in straight rows	Two zones three corridors straight row step type
Furniture design	Unfixed double desks Single-seat	Unfixed double desks Single-seat	Unfixed double desks Single-seat	Fixed desks and row seats (Multiple users)	Fixed desks and row seats (Multiple users)	Fixed desks and row seats (Multiple users)
Technical facilities	Projector	Blackboard Projector Desktop computer Microphone	Blackboard Projector Desktop computer Microphone	Blackboard	Blackboard Projector Desktop computer Microphone	Blackboard Projector Desktop computer Microphone
*Per capita* area	1.77 m^2^ / person	1.89 m^2^ / person	1.33 m^2^ / person	1.01 m^2^ / person	0.95 m^2^ / person	0.82 m^2^ / person
Photo	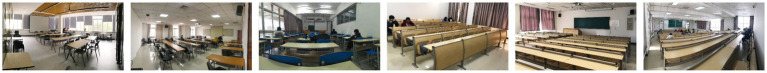					

### Active learning classrooms

In 2018, university H designed and built several active learning classrooms in response to China’s education reform (Ministry of Education of the People’s Republic of China, 2018). Currently, 66 active learning classrooms have been completed and are in use on the main campus, mainly in the West 5th and East 9th Teaching Buildings. University H’s active learning classrooms use sound-insulating wall materials and integrate the classroom lighting, air conditioning, and multimedia systems into a control center at the teacher’s podium for easy management. Furthermore, there are no raised areas in the classroom spaces, and the furniture is designed to be movable to allow students to efficiently study in small groups. Active learning classrooms are equipped with multiple electronic monitors, desktop computers, wireless microphones, mobile whiteboards, and other teaching-friendly equipment to facilitate classroom learning and interactive collaboration among students. University H’s active learning classroom can be classified into four types based on classroom area, functional layout, and furniture design: table and chair integrated, single-user spliceable combination, multi-user spliceable combination, and multi-user fixed combination modes (refer to Table 2).

**Table 2:** Current status of active learning classroom space at university H.

Model	Table and chair integrated	Single-person spliceable combination	Multi-user spliceable combination	Multi-user fixed combination
Size	30 people	42 people	48 people	40 people
Space layout	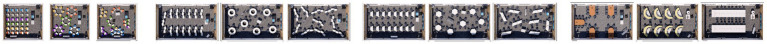			
	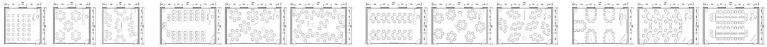			
Furniture design	Colorful movable integrated furniture (with storage area)	Movable fan-shaped desks (400/800*750 mm) Movable seats	Movable trapezoidal desks (700/1370*750 mm) Movable seats	Fixed U-shaped, semicircular, rectangular desks and movable seating (with outlets and storage areas)
*Per capita* area	1.9 m^2^ / person	2 m^2^ / person	1.8 m^2^ / person	2.2 m^2^ / person
Technical Equipment	Display (*4), movable whiteboard, PC, control terminal, microphone	Display (*6), movable whiteboard, PC, control terminal, microphone	Display (*6), movable whiteboard, PC, control terminal, microphone	Display (*6), movable whiteboard, PC, control terminal, microphone
Axonometric drawing	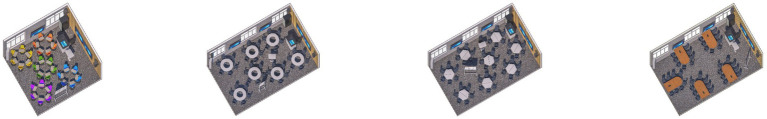			
Photo	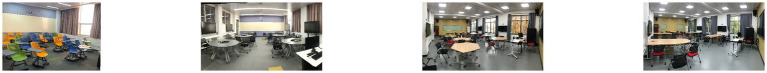			

## Survey methodology

### Survey design

Questionnaires were distributed in lecture and active learning classrooms at university H. The reasons for choosing the lecture and active learning classrooms of this university are as follows. First, lecture classrooms at university H have been constructed and developed for an extended period and are of full scale and size, which reflects the general characteristics of traditional lecture classrooms in Chinese universities. Second, active learning classrooms at university H have been in use for 4 y and are all laid out spatially based on the design principles of SCALE-UP. Some technological improvements and furniture design were conducted in conjunction with the development of Chinese education.

The target population of this study was undergraduate, graduate, and doctoral students attending classes on the main campus of university H. As the university has lecture and active learning classroom courses at various academic stages, participating students have had the opportunity to experience various classrooms. This study was conducted during the second half of the 2021 academic year. The questionnaires were distributed in two formats: online through a student classroom network group and face-to-face in classrooms for 1 month. Informed consent was obtained from all students before the survey experiment.

### Questionnaire design

The questionnaire used in this study was divided into two sections. The questionnaire used in this study was divided into two sections (refer to Appendix). The first section required students to provide personal information, such as their sex, academic stage, and discipline. This demographic information can be used as control variables in regression analysis to ensure that the regression model is not affected by students’ personal information. The second section was divided into two parts: lecture and active learning classroom spaces. Answers were rated on a 5-point Likert scale. The dimensional design of the two classroom space satisfaction scales was based on the exploratory factor analysis of the Spatial Factors Affecting Students’ Learning Experience in Classrooms questionnaire conducted by Peng et al. (2022). This questionnaire divided classroom space factors into four dimensions (instructional interaction, furniture perception, learning support, and physical environment) and found that all four dimensions significantly affected students’ learning experience. This study drew on that mature scale to analyze students’ spatial satisfaction. In addition, the design and preparation of the questions for each dimension simultaneously incorporated the spatial elements that exist in the current state of classrooms at university H. The final student satisfaction with lecture classroom space scale consisted of 19 questions, including five on instructional interaction, four on furniture perception, three on learning support, and seven on the physical environment. Student satisfaction with active learning classroom space scale consisted of 22 questions, including eight on instructional interaction, four on furniture perception, three on learning support, and seven on the physical environment.

## Results

### Current status of student satisfaction with classroom spaces

Statistical analyses were conducted using SPSS 24. A total of 350 questionnaires were distributed, and 341 were returned. After eliminating incomplete, duplicate, or invalid responses, 316 valid responses were used in the analysis (refer to Table 3). The sample size distribution was consistent with the current distribution of students in the university. The Cronbach’s alpha of the scales of student satisfaction was 0.945 and 0.965 for lecture classrooms and active learning classrooms, respectively. The alpha for both scales was higher than 0.9, which indicates that the reliability of the student satisfaction scale data was excellent in the two classroom environments.

**Table 3:** Participant characteristics (*n* = 316).

Sex	Number of people	Percentage
Male	228	(72.1%)
Female	88	(27.9%)
Academic stage	Number of people	Percentage
Undergraduate	260	(82%)
Master’s degree	35	(11%)
PhD	20	(6.3%)
Other	1	(0.7%)
Professional disciplines	Number of people	Percentage
Philosophy, Economics, Law	6	(1.9%)
Education, Literature, History	15	(4.7%)
Science, Engineering, Agriculture, Medicine	290	(91.7%)
Military science, Management, Art	5	(1.7%)

**Figure 2:**
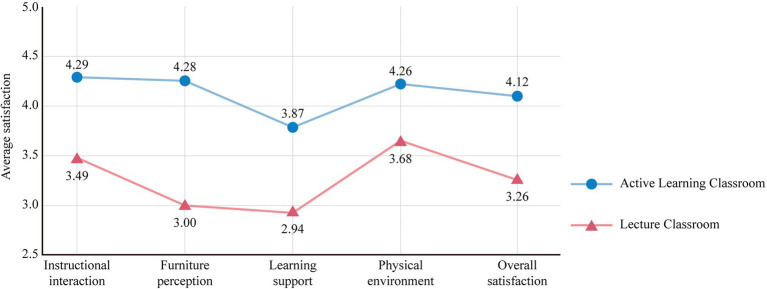
Comparison of student satisfaction between lecture and active learning classroom space.

Figure 2 shows the results of descriptive statistics for the student satisfaction scales in the two types of classroom environments. For lecture classrooms, the mean value of overall satisfaction was 3.26 out of 5, where students were most satisfied with the physical environment, followed by instructional interaction and furniture perception. Students were most dissatisfied with learning support. For active learning classrooms, the mean value of overall satisfaction was 4.12 out of 5, where students were most satisfied with instructional interaction, followed by furniture perception and physical environment. Students were most dissatisfied with learning support.

Overall, satisfaction with all spatial dimensions of active learning classrooms was higher than in lecture classrooms. Furniture perception demonstrated the largest difference in satisfaction, followed by learning support and teaching interaction, whereas the physical environment showed the least difference. Furthermore, learning support was the most unsatisfactory spatial factor for both lecture and active learning classrooms.

### Exploratory analysis of spatial factors affecting student satisfaction in classrooms

In this study, the Kaiser-Meyer-Olkin (KMO) values of student satisfaction were 0.929 and 0.957 for lecture classrooms and active learning classrooms, respectively. Bartlett’s spherical test with *p* = 0.000 < 0.05 for both scales. Tests of the validity of the two scales demonstrated that both scales were suitable for factor analysis.

Table 4 shows the spatial factorization of the student satisfaction with lecture space scale after exploratory factor analysis and re-clustering. After several explorations, two question items were deleted, and three factors with eigenvalues greater than one were obtained, with a cumulative contribution of 65.577% of the explainable variance. According to the initial dimension design of the scale, factor 1 was related to spatial attributes, *per capita* area, table and chair attributes, and user-friendly facilities of the lecture room; therefore, factor 1 was named space and furniture perception (TC_F1). Factor 2 was related to the sound, light, thermal, and decorative environments of the lecture room; therefore, factor 2 was named physical and decorative environment (TC_F2). Factor 3 was related to the display equipment and clarity of field of view of the lecture room; therefore, factor 3 was named visual and pedagogical display (TC_F3).

**Table 4:** The division of spatial factors of lecture classrooms after re-clustering.

Factor	Question item	1	2	3
Space and Furniture Perception TC_F1	Usable area of tables and chairs	0.836		
	The flexibility of use of tables and chairs	0.824		
	Area per person in the classroom	0.806		
	Comfortable use of tables and chairs	0.794		
	Space flexibility in the classroom	0.718		
	Storage space in the classroom	0.717		
	Space comfort in the classroom	0.698		
	Power outlets in the classroom	0.679		
Physical and decorative environment TC_F2	Artificial lighting in the classroom		0.779	
	Temperature and humidity in the classroom		0.770	
	Ventilation in the classroom		0.756	
	Natural lighting in the classroom		0.736	
	Classroom decoration style		0.666	
	The color scheme in the classroom		0.648	
	Sound insulation in the classroom		0.550	
Visual and pedagogical display TC_F3	Range of view of the blackboard in the classroom			0.782
	Clarity of the projector in the classroom			0.756

Extraction method: principal component analysis; Rotation method: Kaiser normalized maximum variance method; the rotation has converged after 6 iterations.

Table 5 shows the spatial factorization of the student satisfaction with the active learning classroom space scale after the exploratory factor analysis and re-clustering. After several explorations, two question items were deleted, and four factors with eigenvalues greater than one were obtained, with a cumulative contribution of 76.050% of the explainable variance. According to the initial dimension design of the scale, factor 1 was related to table and chair properties, spatial properties, and spatial equality of the active learning classroom; therefore, factor 1 was named spatial perception (ALC_F1). Factor 2 was related to the sound, light, thermal, and decorative environments of the active learning classroom; therefore, factor 2 was named physical environment (ALC_F2). Factor 3 was related to the display equipment and interactive equipment of the active learning classroom; therefore, factor 3 was named teaching interaction (ALC_F3). Factor 4 was related to the humanized facilities and storage space of the active learning classroom; therefore, factor 4 was named learning support (ALC_F4).

**Table 5:** The division of spatial factors of active learning classrooms after re-clustering.

Factor	Question item	1	2	3	4
Spatial perception ALC_F1	The flexibility of use of tables and chairs	0.746			
	Comfortable use of tables and chairs	0.737			
	Space comfort in the classroom	0.728			
	Usable area of tables and chairs	0.722			
	Spatial flexibility in the classroom	0.696			
	Spatial diversity in the classroom	0.658			
	Equality of space layout in the classroom	0.572			
Physical environment ALC_F2	Ventilation in the classroom		0.780		
	Natural lighting in the classroom		0.774		
	Artificial lighting in the classroom		0.767		
	Temperature and humidity in the classroom		0.732		
	Classroom decoration style		0.718		
	The color scheme in the classroom		0.709		
	Sound insulation in the classroom		0.560		
Teaching interaction ALC_F3	Movable writing whiteboard in the classroom			0.776	
	Interactive software experience in the classroom			0.727	
	The use of multi-screen monitors in the classroom			0.701	
	Clarity of electronic displays in the classroom			0.571	
Learning support ALC_F4	Power outlets in the classroom				0.774
	Storage space in the classroom				0.757

Extraction method: principal component analysis; Rotation method: Kaiser normalized maximum variance method; the rotation has converged after 8 iterations.

### Critical spatial elements affecting student satisfaction in two types of classrooms

To explore the relationship between spatial factors and overall satisfaction, bivariate correlation analyses were conducted between the three spatial factors and overall satisfaction after re-clustering for lecture classrooms, and between the four spatial factors and overall satisfaction after re-clustering for active learning classrooms. There was a significant correlation for all variables between the two types of classrooms (refer to Tables 6 , 7).

**Table 6:** Correlation between lecture classroom spatial factors and overall satisfaction.

	TC_F1	TC_F2	TC_F3	TC_OS
TC_F1	1			
TC_F2	0.658^**^	1		
TC_F3	0.595^**^	0.596^**^	1	
TC_OS	0.693^**^	0.628^**^	0.533^**^	1

** Means at the 0.001 level (two-tailed), the correlation is extremely significant; TC_F1 = Space and furniture perception, TC_F2 = Physical and decorative environment, TC_F3 = Visual and pedagogical display, TC_OS = Overall satisfaction of lecture classroom.

**Table 7:** Correlation between active learning classroom spatial factors and overall satisfaction.

	ALC_F1	ALC_F2	ALC_F3	ALC_F4	ALC_OS
ALC_F1	1				
ALC_F2	0.799^**^	1			
ALC_F3	0.767^**^	0.754^**^	1		
ALC_F4	0.614^**^	0.584^**^	0.574^**^	1	
ALC_OS	0.659^**^	0.609^**^	0.617^**^	0.512^**^	1

** Means at the 0.001 level, the correlation was extremely significant; ALC_F1 = Spatial perception, ALC_F2 = Physical environment, ALC_F3 = Teaching interaction, ALC_F4 = Learning support, and ALC_OS = Overall satisfaction of active learning classroom.

To further identify the critical spatial factors affecting overall student satisfaction in both classrooms and establish regression equations, this study conducted a linear regression analysis between the spatial factors and their overall satisfaction in each of the two classrooms. To control the influence of students’ sex, academic stage, and major discipline on the regression analysis, independent sample t-tests were conducted for sex, and one-way ANOVAs were conducted for academic stage and major discipline. The results revealed that students’ sex had a significant effect on student satisfaction in the lecture classroom. Therefore, sex was utilized as a control variable in the regression analysis between lecture classroom space factors and overall satisfaction.

For lecture classrooms, linear regression analyses were conducted with three classroom space factors as independent variables, overall student satisfaction as the dependent variable, and student sex as the control variable. There was no multicollinearity among the independent variables, and all VIFs were less than 3 (refer to Table 8). The linear regression model fit well, with *R*^2^ = 0.539, indicating that the three lecture classroom space factors collectively explained 53.9% of the variance in overall satisfaction. The regression equation was: Overall Satisfaction = 0.373 + 0.444 × TC_F1 + 0.326 × TC_F2. Space and furniture perception and physical and decorative environment significantly influenced students’ overall satisfaction (*β* = 0.458 and 0.263, respectively; *p* < 0.001).

**Table 8:** Summary of the linear regression between lecture classroom spatial factors and overall satisfaction.

Variables		B	SE	Beta	T	Sig
(Constant)		0.373	0.224		1.664	0.097
TC_F1		0.444	0.053	0.458	8.424	0.000^***^
TC_F2		0.326	0.068	0.263	4.767	0.000^***^
TC_F3		0.105	0.052	0.104	2.032	0.043
Control Variable	Sex	−0.019	0.081	−0.009	−0.239	0.811
*R* = 0.734		*R*^2^ = 0.539	Adjusted *R*^2^ = 0.533,*F* = 90.854^***^			

*** *p* < 0.001.

Variables: (constant); TC_F1 = Space and furniture perception, TC_F2 = Physical and decorative environment, TC_F3 = Visual and pedagogical display; Dependent variable: Overall satisfaction with lecture classrooms.

For active learning classrooms, linear regression was conducted with four active learning classroom space factors as independent variables and overall student satisfaction as dependent variables. There was no multicollinearity among the independent variables, and the VIF was all less than 4 (refer to Table 9). The linear regression model fit well, with *R*^2^ = 0.480, indicating that the four active learning classroom spatial factors collectively explained 48% of the variance in their overall satisfaction. The regression equation was: Overall Satisfaction = 0.844 + 0.349 × ALCs_F1. Spatial perception significantly influenced students’ overall satisfaction (*β* = 0.337; *p* < 0.001).

**Table 9:** Summary of the linear regression between active learning classroom spatial factors and overall satisfaction.

Variables	B	SE	Beta	T	Sig
(Constant)	0.844	0.203		4.155	0.000^***^
ALC_F1	0.349	0.079	0.337	4.391	0.000^***^
ALC_F2	0.129	0.079	0.120	1.623	0.106
ALC_F3	0.208	0.072	0.199	2.872	0.004
ALC_F4	0.088	0.039	0.122	2.280	0.023
*R* = 0.693	*R*^2^ = 0.480	Adjusted *R*^2^ = 0.473, *F* = 73.729^***^			

*** *p* < 0.001.

Variables: (constant); ALC_F1 = Spatial perception, ALC_F2 = Physical environment, ALC_F3 = Teaching interaction, ALC_F4 = Learning support; Dependent variable: Overall satisfaction with active learning classrooms.

## Discussion

The four dimensions of “physical environment,” “teaching interaction,” “furniture perception” and “learning support” affect students’ learning experience in higher education classrooms. Through the satisfaction evaluation, the degree of difference between students’ expectation level and the actual classroom environment can be compared, in order to put forward certain strategies for the improvement of classroom learning perception in the future. This study employed traditional lecture classrooms and active learning classrooms in university H as the research objects and explored the critical elements affecting students’ spatial perception in the classroom environment through the statistical analysis of the questionnaire. The results are as follows:

1. Students were most satisfied with the “physical environment” of lecture classrooms and “instructional interaction” of active learning classrooms, whereas the “learning support” in both classrooms was the least satisfactory element.
2. Student satisfaction with active learning classrooms was significantly higher than with lecture classrooms, with the most notable differences in “furniture perception” and the least notable difference in “physical environment.”
3. “Space and furniture perception” and “physical and decorative environment” were the critical elements in improving students’ overall satisfaction in lecture classrooms, and “space perception” was the critical element in enhancing students’ overall satisfaction in active learning classrooms.

### Students were most satisfied with the physical environment in lecture classrooms and instructional interaction in active learning classrooms

Due to the current increase in student enrolment and limited investment in education funds, the number of lecture classrooms being built and the percentage of campus space occupied are unlikely to change significantly shortly (Report, 2015). In the descriptive statistical results obtained in this study, the student’s perception score of the traditional classroom physical environment dimension is the highest, which is 3.68. The teaching environment of the traditional classroom needs to meet the needs of many students for them to learn knowledge in a relatively limited space; thus, its physical environment has become the main consideration in space design and construction. As the most common educational environment, the physical conditions of the classroom are controlled and approved by relatively strict construction standards and norms in various countries and regions, so students’ spatial perception can be guaranteed when studying in the traditional classroom. Choi (2014) found that elements of the physical environment of the lecture classroom had a positive effect on student satisfaction.

Due to evolving educational concepts of sustainable education (Cebrián et al., 2020), next-generation learning spaces (Wilson and Randall, 2022), and smart teaching environments (Huang et al., 2012), active learning classrooms continue to be updated and iterated in the design and recreation of spaces (Temple, 2008). As active learning classrooms are implemented in universities worldwide, faculty and students are becoming more comfortable with them and realizing their advantages. In the descriptive statistical results obtained in this study, the student’s perception score of active learning classroom teaching interaction dimension is the highest, which is 4.29. During the development of the active learning classroom, the interactive design of the classroom, including display devices, mobile whiteboards, and interactive software, has completely changed the teaching and learning mode of teachers and students, and the learning environment is more student-centered, focusing on students’ active learning, deep learning, and cooperative learning. The new teaching interaction mode has also become the most satisfactory spatial element of the classroom for students in their learning experience. Several studies have found that flexible teaching environments and rich interactive technologies are the key reasons that active learning classrooms are gratifying (Rands and Gansemer-Topf, 2017; Odum et al., 2020) and have a good influence on students (Jeong and González-Gómez, 2021).

### Learning support was the most unsatisfactory element of the classroom space

The traditional lecture classrooms have been under construction and development for nearly two centuries, and they remain the most dominant teaching space in higher education (Gurzynski-Weiss et al., 2015). In the descriptive statistical results obtained in this study, traditional lecture classrooms’ learning support dimension has the lowest perceived score of students, which is 2.94. It is mainly because the design of the teaching environment is intended to serve mostly lecture-based teaching strategies. As construction demand is high and construction conditions are simple, classroom space construction is largely standardized and modularized to meet efficient teaching use (Painter et al., 2013). This efficient construction model frequently disregards students’ human needs, including a lack of storage space due to a large number of students in the same classroom space, a lack of power outlets for electronic devices, such as laptops and smartphones, and a poor network signal due to the overcrowded classroom space. Waltz et al. (2020) suggested that for the design of lecture classrooms, personal storage cabinets and sufficient electrical outlets should be provided to increase students’ spatial satisfaction and motivation in the classroom. This lack of humanized facility design and construction is the current reason for low satisfaction in lecture classrooms, and it should be given significant consideration in future renovation and design of lecture classrooms to provide learning support.

Active learning classroom design and construction principles are based on new learning theories, such as collaborative learning, exploratory learning, and deep learning (Jamieson et al., 2000; Fisher and Newton, 2014), emphasizing diversified and open teaching spaces and adaptable and rich interactive technology. Although active learning classrooms’ flexible space configuration and sophisticated technological equipment have had a positive impact on student learning, there is still a lack of human services in classroom spaces, such as a lack of storage space, insufficient or unevenly distributed power outlets, and unstable network models. In the descriptive statistical results obtained in this study, the student’s perception score of the learning support dimension of the active learning classroom is the lowest, which is 3.87. The main reason is that the environmental design of active learning classroom mainly emphasizes constructivist learning behaviours such as students’ active learning and cooperative learning while ignoring students’ learning experience and use needs. As a result, learning support elements become the most unsatisfactory spatial points in active learning classrooms. Henshaw and Reubens (2014) found that while students rated active learning classrooms positively, they mentioned that the lack of storage space created a negative experience in active learning classrooms (Henshaw and Reubens, 2014). In the future renewal and design of active learning classrooms, emphasis should be placed on the balance between the actual needs of students and the active learning teaching model to meet the new teaching objectives of fostering active learning, cooperative learning, and deep learning, while also considering the needs of students for basic humanized facilities.

### Students were significantly more satisfied with active learning than lecture classrooms

Active learning classrooms are based on lecture classrooms but with improved physical environment facilities, different classroom space layouts and furniture design, and various teaching and learning interactive technologies to enable new teaching and learning paradigms (Cleveland and Fisher, 2013). Among these improvements, the furniture of active learning classrooms has undergone a disruptive change from the original fixed settings, continuous rows of settings or giant desks, and uncomfortable seats that were difficult to move (Shiyan, 2019) to redesigned furniture that is movable, adjustable, and focused on comfort in use (Dori et al., 2003). Some furniture provides special needs, such as combined tables and chairs (Henshaw and Reubens, 2014; Zimmermann et al., 2018; Odum et al., 2021), spliceable combinations (Mcarthur, 2015; Parsons, 2016), and foldable storage (Chiu, 2016), based on the function of active learning classrooms. Therefore, furniture perception in active learning classrooms was the most notable spatial element that influenced student satisfaction. In the descriptive statistical results obtained in this study, the student satisfaction with active learning classrooms’ furniture design increased the most compared with the traditional classroom, reaching 25.6%. This finding was consistent with previous studies that demonstrated that the perception of furniture in active learning classrooms has a greater positive effect on students’ sense of learning than in lecture classrooms (Yang et al., 2013; Lam et al., 2019).

In addition, as a new generation of learning environments, the active learning classroom has significantly improved its physical and decoration environment and facilities. However, in the descriptive statistical results obtained in this study, the student satisfaction with the physical environmental dimension of the active learning classroom has the smallest improvement compared with the traditional classroom, which is 11.6%. The main reason is that the building environment, construction standards, regulations and norms, and other construction conditions of the active learning classroom are not different from those of the traditional classroom, so the student’s perception of the physical environment in the two classrooms—the length, width and height of the classroom, the window size of the classroom, light brightness and light colour, temperature and humidity, and other indoor physical environments—does not change significantly. Furthermore, the classroom, as the space where students have the most contact and perception in the learning experience, is still a part of the educational environment on the university campus. The learning activities carried out by students in the classroom are also affected by the physical conditions outside the classroom. Whether it is a traditional classroom or an active learning classroom, in the design of the classroom environment, the designer should consider the correlation between the physical conditions of the internal environment and the external environment of the space, such as the impact of indoor artificial lighting and natural outdoor lighting, the connection between the indoor spatial layout and the outdoor environmental vision, the indoor decorative facilities and the outdoor landscape. The correlation between these indoor and outdoor physical environments may also play a crucial role in students’ learning experience and perception.

### Space and furniture perception and physical and decorative environment were critical factors in enhancing the satisfaction of the lecture classroom

According to the main learning activities, lecture classrooms can be divided into individual independent learning and instructor-led learning. Individual learning activities involve a highly focused process of internalizing knowledge, whereas instructor-led learning activities focus on the ability to convey knowledge and the sense of the learning domain of knowledge. In the linear regression results of this study, the space and furniture perception in traditional classrooms is one of the most critical factors affecting students’ learning experience, with a beta value of 0.458. First, the teacher-centred design principle of lecture classrooms pursues maximum student capacity and a structured learning order, which has led to an increasing density of space and furniture and a tendency to simplify and standardize space layout and furniture design.

However, the spatial perception of the classroom often needs to be improved and promoted through subjective student behaviour activities and spatial adaptability. Granito and Santana (2016) indicated that the fixed and repetitive arrangement of desks and chairs in lecture classrooms resulted in low space utilization, which affected the students’ learning experience (Granito and Santana, 2016). Therefore, the traditional classroom should pay more attention to students’ learning experience and behavior, change the standard parallel layout to a more space adaptive individual or group layout, and transform the fixed furniture design into a movable furniture mode that can be adjusted according to students’ subjective biological and psychological characteristics. Second, the traditional classroom should increase the inclusiveness and adaptability of the classroom space on the premise of teaching and a high-density learning environment, to maximize student capacity and educational goals, but also provide a certain sense of learning domain and learning richness, which can improve the perceived satisfaction of the traditional classroom. At present, the space of the traditional classroom is relatively single and fixed, and the learning of the disabled is not considered in the design process. Increasing the spatial inclusiveness of the classroom, such as reducing obstacles in the space, adding furniture for the disabled, and configuring more convenient display equipment, can also comprehensively improve the physical and psychological perception of disabled students.

Furthermore, modern education is gradually seeking a more sophisticated and comfortable learning environment and attempting to create a more suitable physical and ornamental learning environment through various materials and equipment (Woolner et al., 2007). In the linear regression results of this study, the physical and decorative environment of traditional classrooms is also one of the most critical factors affecting students’ learning experience, with a beta value of 0.263. It shows that a comfortable and refreshing learning environment can also directly improve students’ satisfaction. As the most intuitive response of students to learning space perception, the physical environment directly affects the biological subjective feelings of learners. Through the control of temperature and humidity in the traditional classroom, the design of ventilation conditions and lighting conditions, and the improvement of artificial lighting devices, students’ space perception can be directly improved. This can also stimulate students’ creativity and learning motivation, thus improving students’ learning effect. Lam et al. (2019) stated that adequate natural light and visibility in classroom spaces could significantly enhance the attractiveness of the space, bring aesthetic pleasure to students, and raise classroom space satisfaction. Woolner et al. (2007) argued that the temperature of the classroom space was an important factor in student satisfaction, along with the thermal environment, such as air quality.

Therefore, future lecture classroom renewal and restoration should prioritize the diverse and situational design of classroom spaces, the comfort and flexibility of furniture use, and the appropriateness and refinement of the physical environment and interior decorating of classroom spaces.

### Spatial perception was the critical spatial factor in enhancing active learning classroom satisfaction

The continuous development and reform of educational science provided new methods and ideas for active learning classrooms’ spatial layout and furniture configuration (Hacisalihoglu et al., 2018). The use of multiple tools and layouts creates flexible and variable conditions to support the space, and the comfortable and durable furniture attributes transform the original single and fixed learning activities of sitting in rows, resulting in diverse collaborative learning and deep learning space environment (Byers et al., 2018a). In the linear regression results of this study, the spatial perception of active learning classroom is the most critical factor affecting students’ learning experience, with a beta value of 0.337. This shows that in the active learning classroom, a more student-centered, flexible, and enriched spatial layout is the most critical factor affecting students’ perception of satisfaction.

First, the design of active learning classroom is more based on student learning, which greatly improves students’ subjective psychological feelings. For example, the *per capita* area of the classroom is larger, and there are fewer fixed devices in the classroom so that students can move more freely. The layout of the classroom is more student-oriented, and students can form independent, or group diversified learning activities. The use area of furniture is increased to give students a more comfortable user experience.

Secondly, flexible spatial layout and mobile facilities increase the adaptability and inclusiveness of the classroom, and form a complementary use mode. For example, furniture design that can be spliced and combined provides the basis for a diversified spatial layout, so that it can accommodate more abundant teaching activities and learning modes. A more flexible and variable space layout can also provide a place for mobile and spliced furniture design. The use of classroom space can vary from person to person, forming a constantly changing and personalized learning environment (Santoianni et al., 2017). Some studies revealed that the spatial layout or furniture and equipment in active learning classrooms substantially impacts student feeling regarding using the classroom, with many users feeling satisfied (Odum et al., 2020; Rogers, 2020; Zhu and Basdogan, 2021). The higher inclusiveness of the active learning classroom also meets the use needs of special learners (Santoianni and Ciasullo, 2017). The accessible classroom space and flexible furniture configuration enable students with physical disabilities to carry out normal learning activities. The environment that is more student-centered and encourages students to cooperate and discuss gives students with psychological problems the opportunity to share their ideas. Baepler found through interviews with disabled students in the active learning classroom that the spatial perception of the classroom played a positive role in the learning process of these disabled students (Baepler, 2021).

Third, the more equal spatial relationship between teachers and students in the spatial perception dimension of the active learning classroom is also a special reason that affects satisfaction. In active learning classrooms, the podium is not delimited in space. The teacher can walk among the students, which brings the teacher and students closer together. Students can talk and communicate with the teacher more frequently (Swinnerton, 2021). In active learning classrooms, teachers are no longer the transmitters of knowledge; rather, the students become the participants and leaders of active learning in the learning space, and the classroom activities are mainly interactions, cooperation, and discussions among students. Active learning classrooms are built on the principle of student-centeredness, and this more egalitarian teacher-student relationship shifts the centre of the classroom toward the student (Smith, 2017), increasing the student’s psychological ownership of the learning space, which can influence student satisfaction with the overall classroom environment.

## Conclusion

Recently, with the vigorous development of the theory and practice of higher education classrooms, its changes and innovations in teaching mode, space design, and educational technology have had a profound impact on the existing learning environment. This study explored the current status of spatial satisfaction and differences between the classroom types based on the student experience perspective. The results identified critical spatial factors that affected overall satisfaction with traditional lecture classrooms and active learning classrooms. These findings can be used to provide focused and forward-looking suggestions for improving lecture classrooms and optimizing active learning classrooms in universities.

In traditional classrooms, space and furniture perception, and the physical and decorative environment are the most critical environmental factors that affect students’ learning experience. Therefore, this study proposes the following seven suggestions for the improvement and optimization of traditional classrooms:

1. Increase the usable area of students’ desks and seats to ensure the comfort of students’ personal learning and group learning.
2. Increase the number and spacing of student corridors in the classroom to ensure the convenience of students in the furniture layout.
3. Adopt seats that can rotate at multiple angles, or the length of desks is set for two people to share, seats and furniture are designed in an unfixed way, and seats for special learners are installed to meet students’ needs for furniture comfort and flexibility.
4. Adopt sound insulation door and window devices, install soft lighting equipment to improve the overall brightness of the classroom, configure air conditioning equipment that can improve air quality and temperature conditions, and ensure that this equipment can be adjusted and controlled in real-time.
5. In the color of classroom walls, choose a more natural tone rather than pure white or gray.
6. Add movable whiteboards or multiple displays. This low-cost, low intervention improvement can increase the interactive ability of the classroom, improve students’ learning satisfaction, and promote classroom participation.
7. WiFi signal devices and power sockets are placed equidistant on the long side of the classroom to provide students with an environment for offline learning and online learning.

For the active learning classroom, the spatial perception of the classroom is the most critical environmental factor affecting students’ learning experience. Therefore, this study proposes the following six suggestions for the future development and improvement of active learning classrooms:

1. Place personal lockers on the side or back of the classroom to provide students with an area where personal items can be placed.
2. Hidden ground sockets are evenly placed on the ground of the classroom to meet the power supply needs of students when using electronic devices for learning.
3. Some WiFi signal enhancement devices can be installed around the classroom to solve the problem of poor network signals caused by too many students in the classroom.
4. Reduce the fixed obstacles in the classroom, so that students can carry out personal learning or cooperative discussion at any position in the classroom.
5. Setting the podium in the center of the classroom can not only increase the interaction between teachers and students but also maintain equality between teachers and students.
6. Increase the usable area of seats and tabletops to improve the comfort of furniture.

In summary, spatial satisfaction with higher education classrooms was closely related to learners’ learning experience. By rethinking and redesigning the critical spatial elements discussed above that affect learning experiences, we can improve students’ classroom space satisfaction and enhance learning experiences in classroom spaces and promote students’ active learning and deep learning ability (Santoianni and Ciasullo, 2017). In addition, the design of higher education classrooms should not only be based on the observations and ideas of researchers and designers, but also strengthen the consideration of the subjective learning experience and perception of space users, and improve the adaptability and inclusiveness of classroom space to meet a more constructive, active and personalized learning method, to improve the success rate of students in mastering their specific disciplines or research fields (Santoianni et al., 2017). However, this study has certain limitations due to our research capacity and time constraints. For example, in terms of research methods, students’ perception of classroom space is only investigated by questionnaire. In the future, qualitative and quantitative methods such as interviews and observations can be used to conduct more comprehensive research on students’ perceptions. In terms of research content, there is a lack of a more comprehensive investigation of the indoor and outdoor environment of the classroom, which can be studied more systematically in the future. In terms of research objects, multiple universities can be added to conduct comprehensive horizontal comparative research. Therefore, these aspects can be further discussed in future research.

## Data availability statement

The original contributions presented in the study are included in the article/supplementary material; further inquiries can be directed to the corresponding author.

## Ethics statement

The studies involving human participants were reviewed and approved by Medical Ethics Committee, Tongji Medical College, Huazhong University of Science and Technology. The patients/participants provided their written informed consent to participate in this study.

## Author contributions

SJ: Writing-original draft, Software, Resources, Investigation, Formal analysis, Data curation, Methodology, Funding acquisition, and Conceptualization. LP: Validation, Project administration, Methodology, Formal analysis, Supervision, Writing-review & editing, and Conceptualization. All authors contributed to the article and approved the submitted version.

## Funding

This research was funded by the National Natural Science Foundation of China, grant number 51978294.

## Conflict of interest

The authors declare that the research was conducted in the absence of any commercial or financial relationships that could be construed as a potential conflict of interest.

## Publisher’s note

All claims expressed in this article are solely those of the authors and do not necessarily represent those of their affiliated organizations, or those of the publisher, the editors and the reviewers. Any product that may be evaluated in this article, or claim that may be made by its manufacturer, is not guaranteed or endorsed by the publisher.

## Footnotes

^1^Constructivist learning theory, proposed by Swiss psychologist J. Piaget in 1966, is a critique and development of the traditional view of teaching and learning. Constructivist learning theory is learner-centered and emphasizes the active exploration of knowledge, active discovery, and active construction of the meaning of what is learned by the learner, rather than hardwiring the educator’s ideas into the educated.

## Appendix: Questionnaire

### Student satisfaction questionnaire regarding classroom space in Huazhong university of science and technology

Hello! Thank you very much for taking the time to fill out this questionnaire. This questionnaire aims to ascertain students’ satisfaction with classroom space and serve as a resource for optimizing classroom space in universities.

1. Your sex is:

**Table:**

A. Male	B. Female

2. Your academic stage is:

**Table:**

A. Undergraduate
B. Master’s degree
C. PhD
D. Other

3. Your professional discipline is:

**Table:**

A. Philosophy, Economics, Law
B. Education, Literature, History
C. Science, Engineering, Agriculture, Medicine
D. Military science, Management, Art

4. How satisfied are you with the following elements of lecture classrooms at university H?

**Table:**

[1 = Very dissatisfied; 2 = Dissatisfied; 3 = Neutral; 4 = Satisfied; 5 = Very satisfied]					
	1	2	3	4	5
Instructional Interaction					
Clarity of the projector in the classroom	□	□	□	□	□
Range of view of the blackboard in the classroom	□	□	□	□	□
Space comfort in the classroom	□	□	□	□	□
Space flexibility in the classroom	□	□	□	□	□
Space size in the classroom	□	□	□	□	□
Furniture Perception					
Area per person in the classroom	□	□	□	□	□
Usable area of tables and chairs	□	□	□	□	□
Comfortable use of tables and chairs	□	□	□	□	□
The flexibility of use of tables and chairs	□	□	□	□	□
Learning Support					
Storage space in the classroom	□	□	□	□	□
Power outlets in the classroom	□	□	□	□	□
WIFI signal in the classroom	□	□	□	□	□
Physical Environment					
Sound insulation in the classroom	□	□	□	□	□
Natural lighting in the classroom	□	□	□	□	□
Artificial lighting in the classroom	□	□	□	□	□
Temperature and humidity in the classroom	□	□	□	□	□
Ventilation in the classroom	□	□	□	□	□
Classroom decoration style	□	□	□	□	□
Colour scheme in the classroom	□	□	□	□	□

5. What is your overall satisfaction with the space in lecture classrooms at university H?

**Table:**

A. Very dissatisfied
B. Dissatisfied
C. Neutral
D. Satisfied
E. Very satisfied

6. How satisfied are you with the following elements of active learning classrooms at university H?

**Table:**

[1 = Very dissatisfied; 2 = Dissatisfied; 3 = Neutral; 4 = Satisfied; 5 = Very satisfied]					
	1	2	3	4	5
Instructional Interaction					
Clarity of electronic displays in the classroom	□	□	□	□	□
The use of multi-screen monitors in classrooms	□	□	□	□	□
Movable writing whiteboard in the classroom	□	□	□	□	□
Interactive software experience in the classroom	□	□	□	□	□
Space comfort in the classroom	□	□	□	□	□
Spatial flexibility in the classroom	□	□	□	□	□
Spatial diversity in the classroom	□	□	□	□	□
Equality of space layout in the classroom	□	□	□	□	□
Furniture Perception					
Area per person in the classroom	□	□	□	□	□
Usable area of tables and chairs	□	□	□	□	□
Comfortable use of tables and chairs	□	□	□	□	□
The flexibility of use of tables and chairs	□	□	□	□	□
Learning Support					
Storage space in the classroom	□	□	□	□	□
Power outlets in the classroom	□	□	□	□	□
WIFI signal in the classroom	□	□	□	□	□
Physical Environment					
Sound insulation in the classroom	□	□	□	□	□
Natural lighting in the classroom	□	□	□	□	□
Artificial lighting in the classroom	□	□	□	□	□
Temperature and humidity in the classroom	□	□	□	□	□
Ventilation in the classroom	□	□	□	□	□
Classroom decoration style	□	□	□	□	□
Colour scheme in the classroom	□	□	□	□	□

7. What is your overall satisfaction with the space in active learning classrooms at university H?

**Table:**

A. Very dissatisfied
B. Dissatisfied
C. Neutral
D. Satisfied
E. Very satisfied

8. Do you have any other suggestions for the classrooms at university H? Do you have any other ideas or feedback?
